# Genetic determinants of amidating enzyme activity and its relationship with metal cofactors in human serum

**DOI:** 10.1186/1472-6823-14-58

**Published:** 2014-07-15

**Authors:** Eric D Gaier, Alison Kleppinger, Martina Ralle, Jonathan Covault, Richard E Mains, Anne M Kenny, Betty A Eipper

**Affiliations:** 1 Department of Neuroscience, University of Connecticut Health Center, 06030 Farmington, CT, USA; 2 Center on Aging, University of Connecticut Health Center, 06030 Farmington, CT, USA; 3 Department of Biochemistry and Molecular Biology, Oregon Health & Science University, 97239 Portland, OR, USA; 4 Department of Psychiatry, University of Connecticut Health Center, 06030 Farmington, CT, USA

**Keywords:** PAM, Neuropeptide, Copper, SNP

## Abstract

**Background:**

α-amidation is a final, essential step in the biosynthesis of about half of all peptide hormones and neurotransmitters. Peptidylglycine α-amidating monooxygenase (PAM), with enzymatic domains that utilize Cu and Zn, is the only enzyme that catalyzes this reaction. PAM activity is detected in serum, but its significance and utility as a clinical biomarker remain unexplored.

**Methods:**

We used well-established enzymatic assays specific for the peptidylglycine-α -hydroxylating monooxygenase (PHM) and peptidyl-α-hydroxyglycine α-amidating lyase (PAL) domains of PAM to quantify amidating activity in the sera of 144 elderly men. Relationships between PHM and PAL activity and serum levels of their respective active-site metals, Cu and Zn, were analyzed. Study participants were also genotyped for eight non-coding single nucleotide polymorphisms (SNPs) in *PAM*, and relationships between genotype and serum enzyme activity and metal levels were analyzed.

**Results:**

Serum PHM and PAL activities were normally distributed and correlated linearly with each other. Serum PAL activity, but not serum PHM activity, correlated with serum Cu; neither activity correlated with serum Zn. Study subjects possessing the minor alleles for rs32680 had lower PHM and PAL activities, and subjects with minor alleles for rs11952361 and rs10515341 had lower PHM activities.

**Conclusions:**

Our results characterize large variation in serum amidating activity and provide unique insight into its potential origin and determinants. Common non-coding polymorphisms affect serum amidating activity and Cu levels. Serum amidating activity should be explored as a biomarker for functionality in the elderly and in additional study groups.

## Background

Peptides are ancient signaling molecules, with roles in plants and animals. Despite their diversity, many secreted peptides share a common biosynthetic pathway. Carboxy-terminal α-amidation is a final and essential step in the synthesis of about half of bioactive peptides in humans [1,2]. While the list of biologically active peptide products continues to grow, our understanding of the complex network of peptidergic signaling pathways and their clinical relevance remains modest.

Peptidylglycine α-amidating monooxygenase (PAM), an integral membrane protein, is the only enzyme known to catalyze the α-amidation reaction [3,4]. The first part of this two-step reaction, the α-hydroxylation of peptidylglycine, is accomplished by the peptidylglycine α-hydroxylating monooxygenase (PHM: EC1.14.17.3) domain of PAM, which requires Cu [5,6]. The second step, C-N bond cleavage to yield the final amidated product plus glyoxylate, is accomplished by peptidyl-α-hydroxyglycine α-amidating lyase (PAL:4.3.2.5), which uses Zn, although several other divalent metals can substitute for Zn. Tissue-specific endoproteolytic cleavage of PAM can produce soluble PHM and PAL, which can be secreted and remain active outside the cell [7,8]. PHM and PAL activity is present in mammalian serum, although the tissue source remains unknown.

The human *PAM* gene contains 25 exons extending over 160 kb at chromosome 5q21.1. Alternative splicing generates at least 5 isoforms [9]. The gene interval shows a low level of recombination and is contained in a single large haplotype block. Little is known about the regulation of *PAM* expression in humans or about potential functional genetic variations in the *PAM* gene. There are nearly 100 annotated coding variants in the human *PAM* gene and their functional consequences have yet to be tested (http://www.ncbi.nlm.nih.gov/SNP/snp_ref.cgi?locusId=5066).

Animal models suggest that one mechanism of regulating PAM expression involves changes in its mRNA stability. In rat models, estrogen down-regulates PAM mRNA in the anterior pituitary via a change in nuclear stability [10]. This effect may be related to changes in the La protein, which binds to a nuclear retention domain in the 3′-untranslated region of PAM mRNA [11]. With regard to this potential regulation by the La protein, we note that the 3′UTR of human *PAM* contains a SNP, rs5855 (A/G, G is the minor, but ancestral, allele), positioned 60 bp upstream of the La protein nuclear retention binding site. The A-allele generates a poly-adenylation consensus sequence (AAUAAA), which is predicted to generate PAM mRNA variants lacking the La protein nuclear retention signal.

Genetic ablation of the gene encoding *Pam* results in embryonic lethality in mice and complete absence of amidating activity [4]. *Pam* heterozygosity (*Pam*^*+/-*^) results in temperature dysregulation, impairments in vasoconstriction, increased susceptibility to seizures, an anxiety-like phenotype, learning and memory deficits and neuronal hyperexcitability in limbic brain structures [12-14]. Interestingly, many of these deficits can be recapitulated in normal mice by mild dietary Cu restriction and are reversed in *Pam*^*+/-*^ mice by dietary Cu supplementation. These observations suggest the presence of a complex, bi-directional relationship between Cu availability and *Pam* expression in mice [15,16] (for review see [17]).

Serum PHM and PAL activity have not been studied in a large human cohort. In this study, we investigate the relationship between serum PHM and PAL activities and serum metal levels in a previously characterized population of elderly men with frailty characteristics [18,19]. We genotyped these men for single nucleotide polymorphisms (SNPs) in non-coding regions of the *PAM* gene and analyzed the relationship of *PAM* genotype with enzyme activities and Cu levels.

## Methods

### Study population

Frail men aged 60 years or older, residing in the community or assisted living, were recruited to participate in the study. The individuals were screened for potential participation in a previously reported study to assess testosterone effects on bone and frailty in men [18] and the relationship between serum Cu and the Cu/Zn ratio with measures of independence and frailty [19]. The data used in this analysis are baseline assessments. All study participants provided written informed consent. Analysis was limited to men due to the hypothesis and design of the original study on testosterone; a similar female cohort was not available for study. The Institutional Review Board at the University of Connecticut Health Center approved the study.

Our sample was of moderate size for the detection of genotype/phenotype correlations. A sample of 120 men with markers having minor allele frequencies of 0.16-0.44 has 80% power to detect large effects (d > 0.7) for the minor allele in recessive genetic models and 80% power to detect medium effects (d = 0.5) using dominant minor allele genetic models. The effect sizes relative to PHM enzyme activity observed for markers rs32680, rs11952361 and rs10515341 under the recessive model were 0.94, 0.57 and 0.84. For marker rs10038600, the observed effect size was 0.38 for the dominant minor allele model. Analysis of larger samples will be important to verify the genotype-phenotype associations observed.

### Biochemical analysis

PHM activity was assayed as described using a trace amount of [^125^I]-Ac-Tyr-Val-Gly, 0.5 μM Ac-Tyr-Val-Gly and 4.0 μM CuSO_4_; serum samples were diluted 10-fold into 20 mM Na TES, pH 7.4, 10 mM mannitol, 1 mg/ml bovine serum albumin, 1% TX-100 (Surfact-Amps X-100) (Thermo Scientific) and 4 μl of the dilution (0.4 μl of serum) was assayed in triplicate in 100 mM Na MES, pH 5.5 [13]. In the absence of exogenous Cu, PHM activity cannot be detected in human serum; based on dose-response curves (data not shown), the addition of exogenous Cu (4.0 μM CuSO_4_) yielded maximal levels of PHM activity for serum samples in both the upper and lower quintiles. PAL activity was assayed in triplicate from the same dilutions (0.2 μl of serum) using a trace amount of [^125^I]-Ac-Tyr-Val-α-hydroxyglycine, 0.5 μM Ac-Tyr-Val-μ-hydroxyglycine, 1 mM CdCl_2_, 0.02% Thesit and 100 mM Na MES, pH 5.5 [20]. Ceruloplasmin was assayed in duplicate using *o*-dianisidine dihydrochloride [21,22]; a linear response was observed with 1.0 to 5.0 μl serum and samples were compared using 2.5 μl serum.

As reported previously [19], inductively coupled plasma mass spectrometry analysis of Cu and Zn were performed using an Agilent 7700x equipped with an ASX 500 autosampler at a radio frequency power of 1550 W, argon plasma gas flow rate of 15 L/min, and argon carrier gas flow rate of 1.04 L/min. Cu and Zn were measured in kinetic energy discrimination mode using He gas (4.3 mL/min). For analysis, serum samples were diluted 25-fold into 1% HNO_3_ (Fisher Scientific). Data were quantified using a 5-point [0-1000 ppb (ng/g)] calibration curve with external standards. For each sample, data were acquired in triplicate and averaged. An internal standard (Er) introduced with the sample was used to monitor for plasma instabilities and correct for changes in sample matrix.

### Genotyping

Selection of markers: There are no validated common (>5%) coding variants in the *PAM* gene. The potentially functional rs5855 SNP upstream of the PAM mRNA La protein nuclear retention binding site was identified using *in silico* inspection of 3′UTR SNPs. Amplification of the 3′-region of PAM mRNA via cDNA copies from human fibroblasts indicated that the A-allele yielded truncated isoforms of PAM mRNA (Jensen and Covault, unpublished). The 3′UTR rs5855 SNP together with 7 TagSNPs identified using the 2008 HapMap CEU population dataset were genotyped using closed-tube fluorescent TaqMan 5′-nuclease allelic discrimination assays. DNA was extracted from peripheral blood samples using a commercial kit (Gentra Puregene, Qiagen, Valencia, CA). Commercial TaqMan assays were used for six markers: rs32680, rs10038600, rs7733485, rs11952361, rs10515341, and rs17296280 [Applied Biosystems Inc. (ABI) Foster City, CA]. Primers and MGB probes were designed using Primer Express v3.0 software (ABI) for 2 SNPs rs249496 (primers: TGGCGCTGGGGCTAGAC and ATGATGACTGACGCGGGTTT; MBG probes: 6-fam-TGCCTTAT*G*ACTCCGGA and vic-TGCCTTAT*C*ACTCCGGA) and rs5855 (primers: TGCCTTTCCTGTTCAGCATTC and TGTCGTCATGTAGCACAAAGTTTCT; MBG probes: 6-fam-CCTGTGGCA*G*TAAA and vic-CTGTGGCA*A*TAAA). Fluorescence plate reads and genotype calls were made using a 7500 Sequence Detection System following PCR amplification for 40 cycles at 95°C for 15 seconds followed by 60°C for 60 seconds. Linkage disequilibrium for the eight SNP markers in this sample of 140 Caucasian men was examined using the software program Haploview v3.2.2 [23].

### Statistical analysis

All variables were checked for normal distribution and the impact of outliers. Normality was tested using simple sample Chi Squared or Kolmogorov-Smirnov tests. Correlation coefficients were used to detect preliminary associations of PHM and PAL activities with serum metals with other measures of interest. Dominant and recessive effects of the minor allele for each SNP were examined using independent groups t-tests comparing either major allele homozygotes with minor allele carriers with major allele homozygotes (dominant minor allele effect model) or minor allele homozygotes vs. major allele carriers (recessive minor allele effect model). Levene’s test was used to guide equal variance assumptions in each comparison. Statistical analyses were performed using SPSS version 22.0.

## Results

### Subject population

One hundred and forty-four community dwelling elderly men were included in this analysis. Baseline information for this sample has been reported previously [19]; their mean age was 77.1 ± 7.6 years and their mean BMI was 26.9 ± 4.4 kg/m^2^. Most men (91%) met the criteria for frailty (18%) or prefrailty (72%). Approximately 50% met criteria for sarcopenia or low muscle mass commonly associated with aging [24]. A complete set of enzyme activity, serum metal measures and genotype information were available for 120 subjects.

### Serum amidating activity

**Figure 1:**
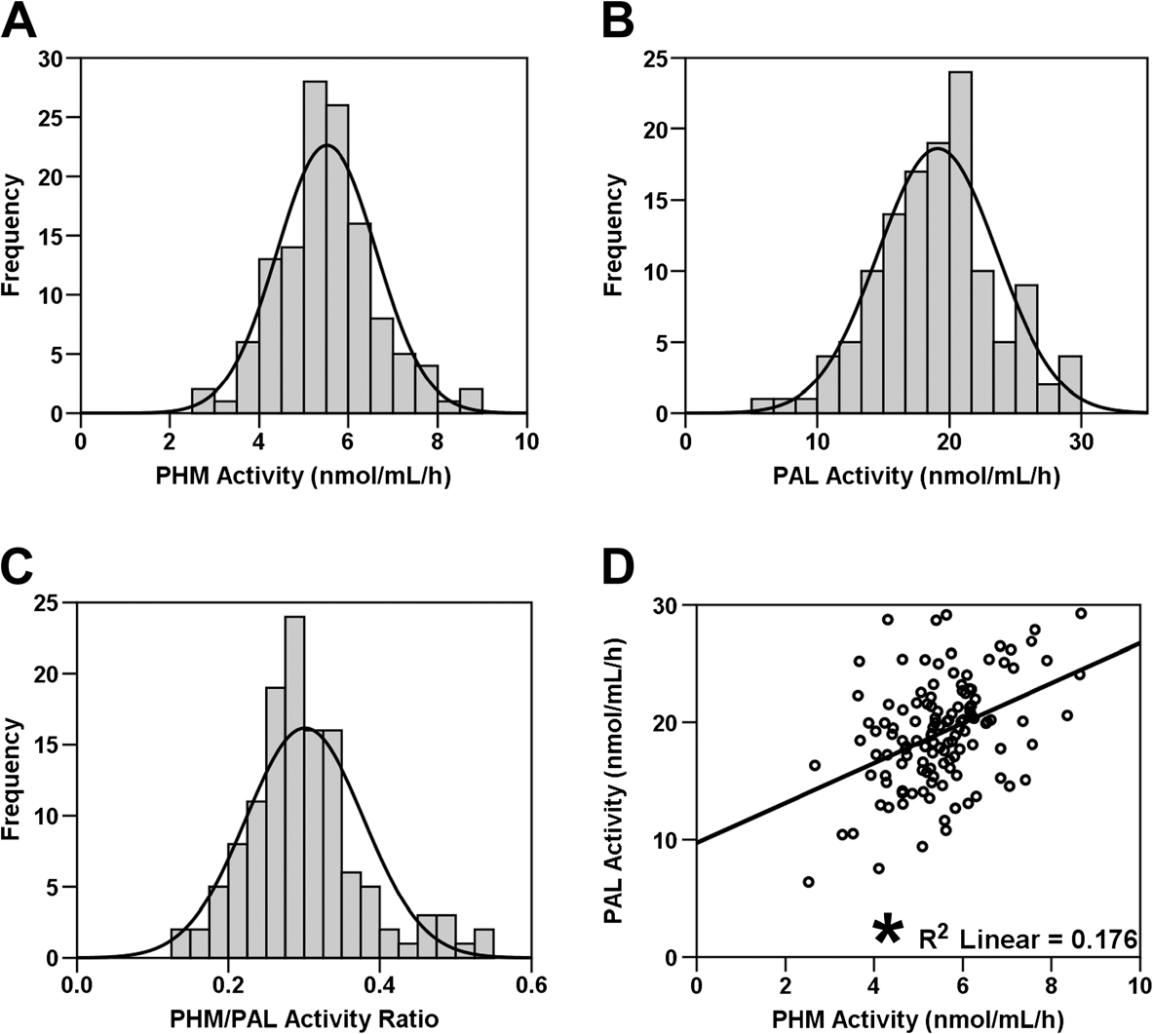
**PHM and PAL Activities.** Graphs depict frequency histograms for PHM **(A)** and PAL **(B)** activities, and the PHM/PAL activity ratio **(C). (D)** PHM and PAL activities for each subject are positively and linearly correlated. *depicts *p* < 0.05 Pearson correlation.

Assays were performed after adding exogenous metals and reflect the amount of PHM and PAL protein, not metallation of each enzyme, in serum [13]. Average serum enzyme activity was 5.51 ± 1.11 nmol/mL/h for PHM and 19.1 ± 4.5 nmol/mL/h for PAL (Table 1). Serum PHM and PAL activities, and the PHM/PAL ratio were normally distributed (Figure 1A,B,C). PHM and PAL activities were significantly correlated (*p* < 0.05) (Figure 1D), as would be expected for enzymes cleaved from the same bifunctional precursor. The average ratio of PHM to PAL activity in serum was 0.30 ± 0.01 (Table 1). Assayed under similar conditions, with the peptide substrate concentration well below K_M_, purified bifunctional PAM-3 yielded a PHM/PAL activity ratio of 0.2 when intact and 0.6 to 0.8 after proteolytic cleavage [25]. Consistent with these observations, when assayed under similar conditions, purified PHM was several fold less active than purified PAL [20].

**Table 1:** Serum amidating activity: descriptive statistics

**Enzyme**	**Mean**	**Standard error**	**Standard deviation**	**Variance**	**Skewedness**	**Kurtosis**
**PHM**	5.51	0.10	1.11	1.24	0.24 ± 0.22	0.71 ± 0.43
**PAL**	19.1	0.4	4.5	20.3	-0.08 ± 0.22	0.14 ± 0.43
**PHM/PAL**	0.30	0.01	0.78	0.01	0.92 ± 0.22	1.34 ± 0.43

### Relationship with serum metals

**Figure 2:**
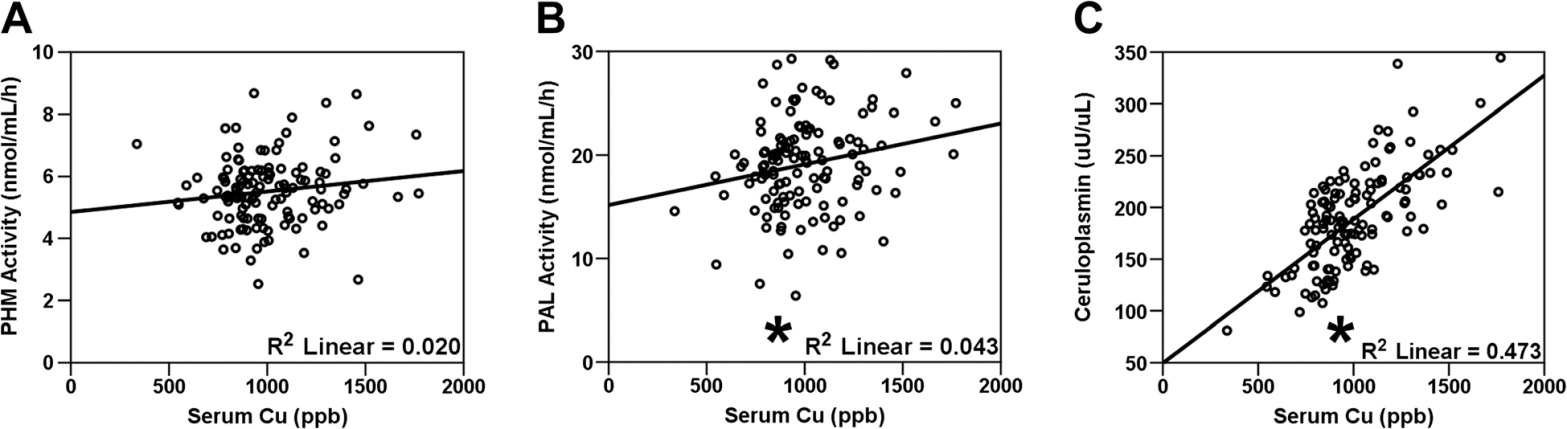
**Serum amidating enzyme activities and metals.** Scatter plots depict PHM **(A)**, PAL **(B)** and ceruloplasmin **(C)** activities versus serum Cu. *depicts *p* < 0.05 Pearson correlation.

Since PHM and PAL utilize Cu and Zn for their enzymatic activities and are co-released from secretory vesicles, we investigated the relationship between PHM or PAL activity and serum Cu and Zn levels. Pearson correlation analyses revealed a significant linear relationship between serum Cu and PAL activity, but not PHM activity (*p* < 0.05) (Figure 2A,B). Serum Cu and ceruloplasmin activity correlated strongly and directly, as expected (Figure 2C). Serum Zn did not correlate with PHM, PAL or ceruloplasmin activity (data not shown).

In our previous study of this same cohort [19], we found significant relationships between the serum Cu/Zn ratio with various measures of health. Similar to the data for serum Cu, the Cu/Zn ratio correlated significantly with PAL activity (R^2^ = 0.023) and ceruloplasmin activity (R^2^ = 0.331), but not with PHM activity (data not shown). Consistent with these relationships, subjects in the highest quintile as sorted by serum PAL activity had significantly higher Cu levels compared to the middle quintile (*p* values < 0.05, data not shown). Therefore, serum PAL activity may serve as an indicator of physical and overall health in our study population.

### SNP analysis

**Figure 3:**
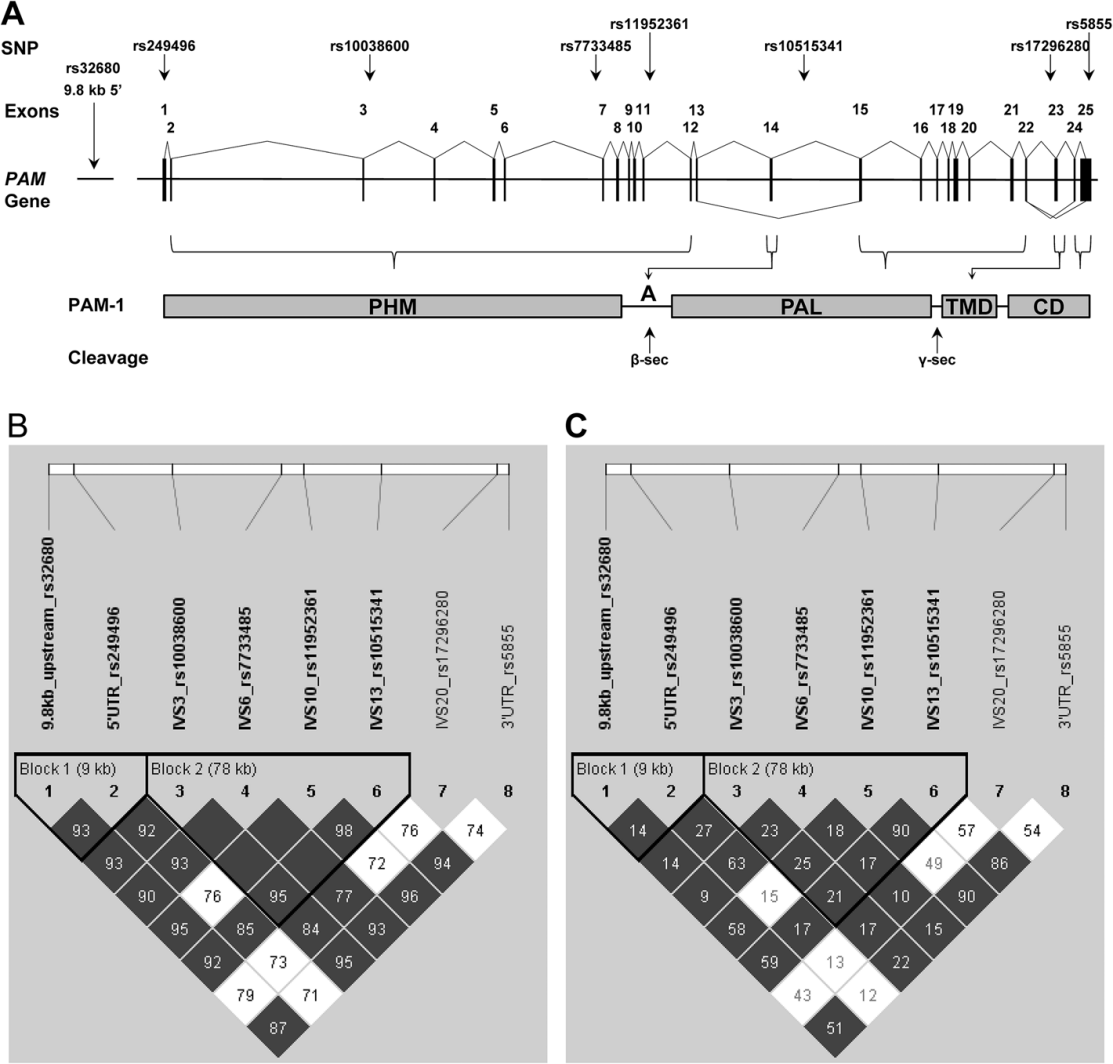
**SNPs in *PAM*. (A)** Schematic of the *PAM* gene with loci of SNPs examined in this study. **(B,C)** Haploview LD plot for eight *PAM* gene SNPs with haplotype blocks identified using the four-gamete rule [26]. Darkened blocks indicate SNP pairs without evidence of extensive recombination (i.e. 4-gamete having a frequency < 0.01). **(B)** LD plot showing D’ - Values shown represent 100 × |D’|, empty boxes represent value of 100 (i.e. |D’| = 1). **(C)** Haploview LD plot showing R^2^ correlation values.

The large variation we observed in serum PHM and PAL activities could arise from genetic variation in and/or regulation of the *PAM* gene (Figure 3A) among our subjects. To address this question, we focused on eight common SNPs (minor allele frequencies 16-44%) (Table 2). In addition to the 3′ UTR rs5855 SNP, a set of seven haplotype TagSNPs (rs32680, rs249496, rs10038600, rs7733485, rs11952361, rs10515341, rs17296280) which had been identified from the 2008 HapMap CEU population dataset (Table 2; Figure 3) were examined. This set of eight markers provides correlation with the larger set of genotyped SNPs in the HapMap dataset in this region with an average R^2^ = 0.925. The haploview pairwise marker linkage disequilibrium (D’) and correlations (R^2^) are illustrated in Figure 3B and C, respectively. Two blocks of SNPs showing very limited recombination are evident: block 1 (rs32680, rs249496) and block 2 (rs10038600, rs7733485, rs11952361, rs10515341).

**Table 2:** ***PAM *** **SNPs studied**

**SNP**	**Ch5 position**	**Alleles**	**GERP**	**Location**	**MAF**
**rs32680**	102191715	C:T	-1.99	9.8 kb-5′	0.159
**rs249496**	102201590	G:C	3.31	5′UTR	0.441
**rs10038600**	102238502	G:T	-0.469	IVS3	0.225
**rs7733485**	102279540	A:G	3.42	IVS6	0.271
**rs11952361**	102287920	A:G	-0.47	IVS10	0.360
**rs10515341**	102317117	G:A	-7.79	IVS13	0.274
**rs17296280**	102360747	A:C	-0.328	IVS20	0.270
**rs5855**	102365186	A:G	5.93	3′UTR	0.326

We compared contrasting minor allele carriers vs. major allele homozygotes (minor allele dominant effect model) or contrasting minor allele homozygotes vs. major allele carriers (minor allele recessive model) for each SNP for serum amidating activity and Cu/ceruloplasmin levels (Table 3). Four SNPs were associated with differences in serum amidating activity. The upstream rs32680 minor allele homozygotes had significantly lower serum PHM and PAL activities (t = -2.38; *p* = 0.019 and t = -2.25; *p* = 0.026, respectively), without a significant difference in the PHM/PAL ratio. The minor T allele at this locus may lower PAM enzyme activity through reduced expression of the PAM gene. The 5′ UTR SNP rs249496 in the same block (block 1), showed no such differences in serum amidating activity. Lower PHM activities were present in the serum of subjects homozygous for the major G allele at the rs10038600 locus in intron 3 (t = -2.07; *p* = 0.041), homozygote subjects for the minor G allele at rs11952361 in intron 10 (t = -2.01; *p* = 0.047), and the minor but ancestral A allele at rs10515341 in intron 13 (t = -2.89; *p* = 0.005), all of which are in the same haplotype block (block 2). Although PAL activity followed a similar trend, none of the associations was significant. PHM/PAL ratios were not different among these genotypes for any of these SNP loci, suggesting that any potential regulatory effects of these SNPs do not selectively influence expression of either enzyme.

**Table 3:** ***PAM *** **SNP genotype relationships with serum PHM and PAL activity and metals**

**SNP**	**rs32680**			**rs249496**			**rs10038600**			**rs7722485**			**rs11952361**			**rs10515341**			**rs17296280**			**rs5855**		
**Geno-type**	**CC (73)**	**CT (39)**	**TT (8)**	**CC (18)**	**CG (48)**	**GG (54)**	**GG (50)**	**GT (52)**	**TT (18)**	**AA (64)**	**AG (43)**	**GG (13)**	**AA (60)**	**AG (45)**	**GG (15)**	**AA (13)**	**AG (46)**	**GG (61)**	**AA (61)**	**AC (47)**	**CC (12)**	**AA (60)**	**AG (47)**	**GG (13)**
**PHM**	**5.57±0.97§**	**5.69±1.25§**	**4.68±1.13§†‡**	5.54±1.02	5.56±1.03	5.54±1.19	**5.31±1.05†‡**	**5.78±1.14§**	**5.56±1.00**	5.58±1.24	5.58±0.91	5.32±0.96	**5.64±1.01**	**5.60±1.09**	**5.03±1.36‡**	**4.74±0.99§†‡**	**5.69±1.16§**	**5.62±1.00§**	5.55±0.97	5.66±1.21	5.11±1.20	5.65±1.03	5.54±1.06	5.10±1.43
**PAL**	**19.3±4.2§**	**19.7±4.6§**	**15.9±4.7§†‡**	20.2±5.0	19.6±3.9	18.5±4.6	19.2±4.5	19.4±4.6	18.6±3.7	18.7±4.6	19.8±3.8	19.6±5.3	19.3±4.1	19.5±5.0	18.0±4.0	17.1±3.7	19.5±5.0	19.4±4.1	19.3±4.2	19.4±4.7	17.8±4.7	19.4±4.2	19.4±4.8	17.7±4.1
**PHM/PAL**	0.30±0.08	0.30±0.07	0.32±0.11	0.29±0.08	0.29±0.07	0.31±0.08	0.29±0.08	0.31±0.08	0.31±0.09	0.31±0.09	0.29±0.06	0.29±0.10	0.31±0.08	0.30±0.07	0.29±0.09	0.29±0.09	0.30±0.07	0.30±0.08	0.30±0.08	0.30±0.07	0.30±0.09	0.30±0.08	0.30±0.07	0.30±0.09
**Cp**	186±44	194±49	192±49	**207±47§**	**195±43**	**178±46§‡**	**196±51§**	**192±41§**	**160±32§†‡**	182±45	197±49	198±34	**183±38†**	**202±51§**	175±52	**168±50†**	**198±48§**	186±42	190±44	191±49	173±41	186±38	195±53	180±51
**Cu**	996±209	1013±256	975±302	**1076±218§**	**1027±236**	**951±221§‡**	**1045±254§**	**984±223**	**921±144§‡**	981±226	1020±248	1030±188	989±212	1020±225	986±314	946±308	1023±232	994±212	989±195	1020±267	980±256	998±210	1002±228	1003±330
**Zn**	846±284	911±263	828±199	879±273	853±283	872±268	903±305	834±237	853±274	871±250	866±321	839±213	840±228	894±339	884±216	885±233	899±332	837±229	822±225	909±331	921±217	842±227	885±336	906±213
**Cu/Zn**	1.26±0.36	1.17±0.37	1.18±0.23	1.31±0.39	1.26±0.29	1.17±0.40	1.23±0.35	1.24±0.37	1.16±0.37	1.19±0.38	1.26±0.36	1.27±0.24	1.22±0.30	1.25±0.44	1.14±0.32	1.09±0.29	1.24±0.42	1.24±0.32	1.26±0.34	1.20±0.39	1.09±0.29	1.23±0.30	1.24±0.43	1.13±0.34
**Cu/Cp**	5.51±1.17	5.31±0.89	5.08±1.00	5.33±1.05	5.38±1.05	5.48±1.12	**5.43±0.97**	**5.22±1.05§**	**5.94±1.30†**	5.51±1.05	5.32±1.19	5.25±0.75	5.54±1.17	5.160.89	5.701.09	5.70±1.17	5.24±0.88	5.50±1.18	5.36±1.14	5.43±1.00	5.71±1.02	5.50±1.17	5.27±0.95	5.57±1.07

The number of subjects (120 total) of each genotype is shown in parenthesis.

Haplotype blocks are indicted by bold borders.

±SD.

Versus 1 OR 3 *p*<0.05 (*t*-test)^**§**^.

Versus 2 *p*<0.05 (*t*-test)^**†**^.

Versus 1 and 2, OR 2 and 3*p*<0.05 (*t*-test)^**‡**^.

With respect to serum Cu and ceruloplasmin, significantly lower serum Cu was found in subjects homozygous for the major G-allele at the rs249496 locus in the 5′ UTR (t = -2.43; *p* = 0.016) and for subjects homozygous for the minor T-allele at the rs10038600 locus in intron 3 (Table 3). Consistent with the tight association between Cu and ceruloplasmin (Figure 2C), serum ceruloplasmin followed the same associations for these SNP genotypes. Interestingly, the Cu/ceruloplasmin ratio, a measure of relative un-bound serum Cu, was elevated in homozygous subjects for the minor T-allele at rs10038600 (t = 2.29; *p* = 0.024).

## Discussion

In the current study, we characterized the distribution of the two activities essential for serum amidating activity, identified relationships between both enzyme activities and their metal cofactors, and found several associations between TagSNP polymorphisms and serum amidating activity and serum Cu in a population of elderly men. This is the first study to examine potential determinants and clinical relevance of the two components of serum amidating activity in human subjects; examination of similar parameters in additional study populations is needed to determine which conclusions can be generalized.

### Serum enzymes and their metal cofactors

Since PHM and PAL are derived from the same gene product in mammals, with PHM requiring Cu and PAL using Zn, assessment of their serum activities and their associations provides insight into the nature and determinants of serum amidating activity. The full-length, integral membrane PAM-1 protein, containing both PHM and PAL domains, is the predominant isoform expressed in mammalian tissues [27]. PHM and PAL are liberated from the transmembrane domain of PAM-1 and made soluble within the regulated secretory pathway by prohormone convertases 1 and/or 2; on the plasma membrane and in the endocytic pathway, other endoproteases, including α-secretase and γ-secretase, can separate PAL from the transmembrane domain and cleave within the PAM transmembrane domain [28]. It is important to note that we added optimal divalent metals to each enzyme assay, so the measured serum activity reflects PHM and PAL protein content without regard to individuals with low serum metal levels. That the PHM and PAL activities were normally distributed, varied among our subjects, and strongly correlated with one another reflects the fact that both are usually produced together as a bifunctional enzyme.

Variation in human serum amidating activity was greater than 20% (Figure 1), exceeding the less than 10% variation observed in inbred mice [12,13]. Mice heterozygous for a knock-out copy of the gene encoding PAM have half the normal levels of serum amidating activity and PAM protein in all tissues studied. These mice display profound physiological and behavioral deficits, reinforcing the importance of having the full complement of PAM [12-14,16]. The degree of variation observed in humans means that our small sample set included individuals with only half the mean value of PHM or PAL, a potentially significant decrease (Figure 1). Over 75 rare (<1%) mutations in the *PAM* gene that could inactivate PHM or PAL or truncate the PAM protein have been annotated in the human genome database (http://www.ncbi.nlm.nih.gov/SNP/snp_ref.cgi?locusId=5066). However, no human disease state has yet to be attributed directly to PAM dysfunction or insufficiency. Sequencing of the *PAM* gene should be considered in patients with deficits that resemble the phenotype of *Pam* heterozygous mice, including temperature dysregulation, metabolic syndrome, anxiety and memory impairments.

Levels of PAL, but not PHM, correlated with serum Cu, and neither activity correlated with serum Zn. Since the major tissue sources of serum PHM and PAL remain unknown, it is difficult to interpret this unexpected result. PHM and PAL bind their respective metal cofactors with relatively low affinity compared to many other metalloenzymes [20,29,30]. Some Cu-dependent enzymes are more stable with Cu bound than when not metallated [31]; this does not appear to be the case for PAM, since neither PHM nor PAL activity correlated with its respective metal co-factor. When cultured pituitary tumor cells expressing membrane PAM were made copper deficient, secretion of PHM increased and endocytic degradation of PAM decreased [32]. Similarly, when C57BL/6 mice were fed a copper deficient diet, serum PHM activity rose [12-14,16]. Although our data suggest that PAM participates in the cell-type specific control of copper homeostasis, we do not yet have a satisfying understanding of the entire system.

### Genetic regulation of serum amidating activity

Several hundred SNPs have been annotated in the *PAM* gene (http://www.ncbi.nlm.nih.gov/SNP/snp_ref.cgi?locusId=5066), many of which code for potentially impactful changes in amino acid sequence or regulation of PAM expression/splicing. The SNPs we examined are common and have no impact on PAM primary structure, yet they showed significant correlations with serum amidating activity and correlate with associated metal levels.

We observed significant minor allele associations with reduced serum PHM activity in block 1 (rs32680) and block 2 (rs11952361 and rs10515341), consistent with a minor allele recessive model for these markers. Significantly reduced PAL activity was found for minor allele homozygotes only at rs32680, with a trend towards reduction for minor allele homozygotes at the two block 2 SNPs (rs11952361 and rs10515341). The concordance of effects for PHM and PAL at these three markers likely reflects their production from the same gene product. Minor splice variants encoding only PHM have been identified [33] and one or more of these SNPs may be involved in regulating such splicing events.These SNPs could affect serum amidating activity through a variety of mechanisms. Up- and down-stream UTR SNPs can alter mRNA stability through microRNA binding. Intron SNPs can affect alternative splicing, which determines whether PAM is an integral membrane or soluble protein and whether an endoproteolytic cleavage site separates PHM from PAL (Figure 3A).

SNPs in both blocks 1 (rs249496) and 2 (rs10038600) were associated with differences in serum Cu. Similar relationships were found for ceruloplasmin, as might be expected given the strong correlation between Cu and ceruloplasmin. At rs10038600, however, homozygosity for the minor T-allele was associated with lower serum Cu and even lower ceruloplasmin as the Cu/ceruloplasmin ratio was elevated in these individuals. Interestingly, homozygosity for the major G-allele at the same locus was associated with lower serum PHM activity, possibly reflecting a dual influence or potential regulatory influence of PHM and Cu homeostasis (see above). This SNP is located at the 5′ end of intron 3, a prime position to influence alternative splicing of exon 3; this could in turn affect Cu binding of PHM. In vitro experiments are necessary to test this hypothesis.

## Conclusions

The tissue sources of serum amidating activity have not yet been identified. While studies of PAM processing and secretion in a pituitary tumor cell line identified a regulatory role for Cu [32], it is not yet possible to extend these in vitro studies to the in vivo situation. The determinants of serum amidating activity are clearly complex and need to be studied as part of the multi-organ Cu homeostasis network now beginning to be elucidated [34]. The data presented here support the idea that manipulation of the *PAM* gene in laboratory models may be a useful tool to study this complex relationship. Additionally, serum amidating activity may serve as a biomarker for certain disease states, including measures of frailty and physical health in the elderly and in Cu deficiency.

## Competing interests

The authors declare that they have no competing interests.

## Authors’ contributions

All authors worked together to conceive of the study. AK and AMK collected the original data and serum samples. JC carried out the genetic studies and drafted the corresponding portions of the manuscript. MR performed all serum metal measurements. EDG performed PAM enzyme assays, did the statistical analyses and drafted the manuscript. EDG, BAE, REM and JC participated in study design and coordination to finalize the manuscript. All authors read and approved the final manuscript.

## Pre-publication history

The pre-publication history for this paper can be accessed here:

http://www.biomedcentral.com/1472-6823/14/58/prepub
